# Uropathogenic *Escherichia coli* Biofilms: Antibiotic Pressure and Interaction with Human Neutrophils

**DOI:** 10.3390/ijms26199484

**Published:** 2025-09-28

**Authors:** Irina L. Maslennikova, Irina V. Nekrasova, Marjanca Starčič Erjavec, Nina V. Karimova, Marina V. Kuznetsova

**Affiliations:** 1 Institute of Ecology and Genetics of Microorganisms Ural Branch Russian Academy of Sciences—Perm Federal Research Center Ural Branch Russian Academy of Sciences, 614081 Perm, Russia; nirina5@mail.ru (I.V.N.); mar19719@yandex.ru (M.V.K.); 2 Department of Microbiology and Virology, Ye. A. Vagner Perm State Medical University, 614000 Perm, Russia; 3 Department of Biology, Faculty of Natural Sciences and Mathematics, University of Maribor, 2000 Maribor, Slovenia; 4 Department of Microbiology, Biotechnical Faculty, University of Ljubljana, 1000 Ljubljana, Slovenia; 5 Centralized Clinical Diagnostic Laboratory LLC, 614000 Perm, Russia; karimova.nv@ckdl.ru

**Keywords:** uropathogenic *E. coli* (UPEC), biofilm formation, polymorphonuclear neutrophils (PMNs), ciprofloxacin, apoptosis, sub-MIC, pre-sub-MIC, antibiotics

## Abstract

Uropathogenic *Escherichia coli* (UPEC) is a primary cause of urinary tract infections (UTIs), with recurrent cases often linked to its ability to form biofilms. This study investigated the effects of various antibiotics on UPEC biofilm formation and the subsequent interaction of these biofilms/their supernatants with human neutrophils. We determined the minimum inhibitory concentrations (MIC), minimum bactericidal concentrations (MBC), and biofilm eradication concentrations (MBEC) for ampicillin, gentamicin, chloramphenicol, ciprofloxacin, and levofloxacin. Our results showed an increase in MBEC compared to MBC for all tested antibiotics, confirming the enhanced antibiotic resistance of bacteria in biofilm. We found that sub-MICs of ciprofloxacin, which moderately inhibited planktonic growth, actually stimulated an increase in biofilm biomass. This antibiotic-induced biofilm growth was accompanied by changes in bacterial morphology, including the formation of elongated, filamentous cells, an adaptive stress response. Biofilm-embedded bacteria, but not their supernatants, significantly reduced neutrophil viability, primarily by inducing neutrophil necrosis. The presence of ciprofloxacin during biofilm formation did not fundamentally alter interactions with neutrophils. These findings highlight the importance of studying effects of antibiotic pressure on biofilm formation, underscoring the challenges in antibiotic treatment of UTIs.

## 1. Introduction

Uropathogenic *Escherichia coli* (UPEC) is the leading causative agent of community-acquired and nosocomial uncomplicated and complicated urinary tract infections (UTIs), accounting for 50 to 90% of all cases [1,2]. Recurrent UTIs, defined as having two or more UTIs within a six-month period or three or more UTIs within one year [3], is in 25–82% of cases caused by the same strain as the initial infection [4], which may be due to *E. coli* persistence/survival in biofilms on biotic (epithelium, stones) and/or abiotic (catheters, stents, drains) surfaces [5]. A rise in the prevalence of antibiotic-resistant UPEC strains has been widely documented in recent publications (e.g., [6,7,8]). Furthermore, a meta-analysis performed by Zhao et al. [9] revealed significant correlation between biofilm formation with antibiotic resistance and virulence factors. In addition, the ability of sub-minimum inhibitory concentrations (sub-MICs) of antibiotics to modulate bacterial virulence in vivo has recently been identified, e.g., sub-MICs of antibiotics have been shown to stimulate uropathogens to adhere to and invade urothelial tissue in mice [10,11].

The treatment of UTIs relies on the use of antibiotics such as β-lactams, fluoroquinolones, and trimethoprim–sulfamethoxazole [12]. However, UPEC strains have the ability to acquire resistance to multiple antibiotics, making treatment options more limited and challenging [13]. Interestingly, among human UPEC, nalidixic-acid-, chloramphenicol- and tetracycline-resistant isolates have reduced virulence potential compared to susceptible strains [14]. There is emerging evidence that antibiotics may have off-target effects when used at sub-lethal concentrations [15] and that antibiotics (e.g., ciprofloxacin, amoxicillin, and aminoglycosides) cause significant genetic and phenotypic changes in UPEC strains leading to modulations in the repertoire of bacterial virulence and biofilm formation [16].

The immune response to UPEC is primarily mediated by Toll-like receptors recognizing lipopolysaccharide, flagella, and other structures on the bacterial surface [17,18]. UPEC have the capacity to subvert this immune response of the host by means of actively impacting on proinflammatory signaling pathways or by physical masking immunogenic structures [18]. This ability is significantly enhanced when UPEC form biofilms, as the extracellular polymeric substance acts as a physical barrier, shielding the bacteria from the host immune response [19]. Moreover, the bacteria in biofilms are more resistant to antibiotics than their planktonic counterparts [5]. It is known that minimum biofilm eradication concentrations (MBECs) of antibiotics are much higher than the minimum inhibitory (MIC) and minimum bactericidal concentrations (MBC) of antibiotics [20,21]. Bacterial cells in the biofilm acquire a high level of tolerance to antibiotics through several mechanisms. The exopolysaccharide matrix can block or slow down the penetration of antibiotics; the cells in the outer layer of the biofilm protect other cells from antibiotics; the killing effect of antibiotics in biofilms can be significantly reduced due to the slow growth of bacterial cells inside [22,23].

The aim of this study was to investigate the effects of the sub-MIC (0.5 × MIC), MIC, MBC, and MBEC of ampicillin, gentamicin, chloramphenicol, levofloxacin, and ciprofloxacin on UPEC strain DL82 in biofilm in comparison to planktonic growth and to investigate the effect of this UPEC strain grown in biofilm in the presence of the pre-sub-MIC (0.25 × MIC), sub-MIC (0.5 × MIC), and MIC of ciprofloxacin and their secretions (biofilm supernatants) on human neutrophils.

## 2. Results

### 2.1. The Antibiotic Sensitivity of Studied UPEC Strain DL82

The studied UPEC strain, DL82, was resistant to ampicillin and had high MIC and MBC values for this beta-lactam drug but was sensitive to all other used antibiotics (gentamicin, chloramphenicol, ciprofloxacin, and levofloxacin) (Table 1). For all used antibiotics the MBEC values were higher than MIC and MBC values (Table 1).

### 2.2. Plankton Growth and Biofilm Formation of UPEC Strain DL82 at Different Ciprofloxacin Concentrations

**Figure 1:**
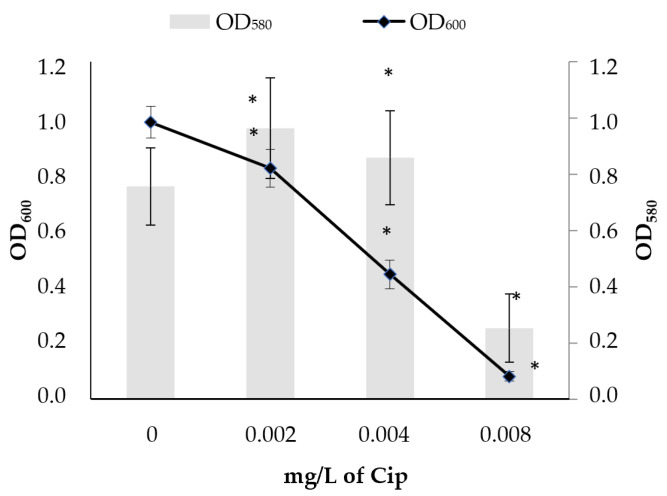
Effect of different ciprofloxacin (Cip) concentrations, pre-sub-MIC (0.25 × MIC, 0.002 mg/L of Cip), sub-MIC (0.5 × MIC, 0.004 mg/L of Cip), and MIC (0.008 mg/L of Cip), on plankton growth and biofilm biomass of UPEC strain DL82. *—statistically significant difference from growth in LB with no added Cip.

In order to evaluate the effect of the pre-sub-MIC, sub-MIC, and MIC of ciprofloxacin on plankton growth and biofilm formation, the strain was grown in LB without and with added ciprofloxacin in different concentrations: pre-sub-MIC (0.25 × MIC, 0.002 mg/L of ciprofloxacin), sub-MIC (0.5 × MIC, 0.004 mg/L of ciprofloxacin), and MIC (0.008 mg/L of ciprofloxacin). As seen from Figure 1 the plankton growth (OD_600_) of *E. coli* strain DL82 was inhibited already at the pre-sub-MIC and sub-MIC (0.002 mg/L and 0.004 mg/L of ciprofloxacin, respectively), while these ciprofloxacin concentrations stimulated formation of biofilm biomass (OD_580_), reaching higher biofilm biomass values than those formed at the MIC (0.008 mg/L). The MIC strongly inhibited not only the plankton growth but also biofilm production.

**Figure 2:**
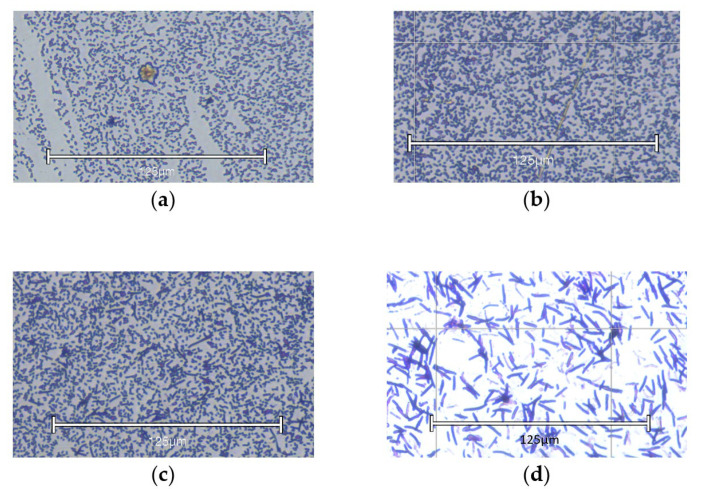
Morphology of UPEC strain DL82 in biofilms grown in sub-MIC, MIC, and 2 × MIC of Cip. EVOS M5000 light microscope, scale bar—125 μm. (**a**) Control—strain grown in LB without Cip; (**b**) strain grown in sub-MIC of Cip (0.004 mg/L); (**c**) strain grown in MIC of Cip (0.008 mg/L); (**d**) strain grown in 2 × MIC of Cip (0.016 mg/L).

To visualize the cell morphology in biofilms, light microscope images of *E. coli* DL82 grown in LB without and with different ciprofloxacin concentrations were obtained (Figure 1). As seen from light microscope images, the increase in biofilm biomass at the sub-MIC (0.5 × MIC) and MIC of ciprofloxacin of *E. coli* DL82 (Figure 1) was not due to changes in cell density of biofilm (Figure 2a–c). Increasing the ciprofloxacin concentration above the MIC to 2 × MIC (0.0016 mg/L) led to a decrease in biofilm biomass and elongation of bacterial cells (Figure 2d).

### 2.3. Effect of Different Antibiotics Supplemented in Pre-Sub-MIC, Sub-MIC, and MIC on Plankton Growth and Biofilm Formation of UPEC Strain DL82

As a difference in growth was observed for ciprofloxacin (Figure 1), the growth tests at the pre-sub-MIC, sub-MIC, and MIC were repeated for all other initially tested antibiotics and a correlation analysis was performed. The correlation analysis of plankton growth and biofilm formation at antibiotic concentrations around the MIC showed that ampicillin and chloramphenicol exposure had similar effects on plankton and biofilm growth (correlation coefficient 0.95 and 0.99, respectively). However, for the two quinolone antibiotics (ciprofloxacin and levofloxacin), the inhibition of plankton growth was accompanied by an increase in biofilm biomass, so the correlation coefficients between plankton growth and biofilm biomass were 0.76 and 0.26, respectively (Table 2).

### 2.4. Neutrophil Viability, Apoptosis, and Necrosis After Interaction of UPEC Strain DL82 Cells and Biofilm Supernatants of Cells Grown in LB Supplemented with Different Ciprofloxacin Concentrations

**Figure 3:**
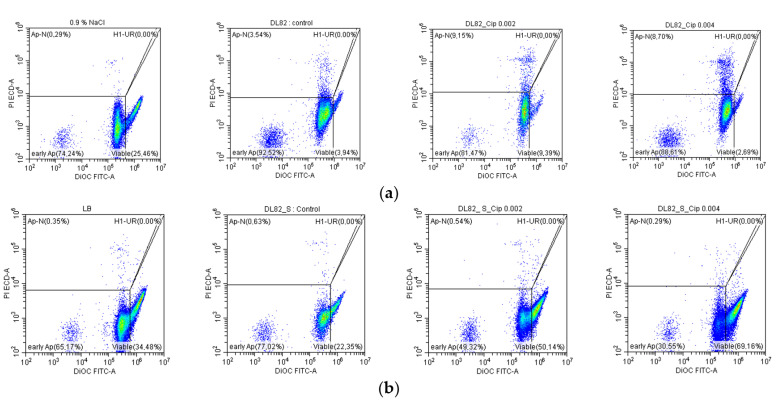
Neutrophil subpopulation after interactions with cells (**a**) and supernatants (**b**) of UPEC strain DL82 biofilms formed under exposure to different concentrations of ciprofloxacin. As seen from Figure 4b, neutrophil viability (DiOC_6_(3)^+^/PI^−^), neutrophil early apoptosis (DiOC_6_(3)^−^/PI^−^), and late apoptosis/necrosis (DiOC_6_(3)^−^/PI^+^) were not changed by *E. coli* DL82 biofilm supernatants compared to the control regardless of the presence of the pre-sub-MIC and sub-MIC of ciprofloxacin in the medium (Figure 4).

**Figure 4:**
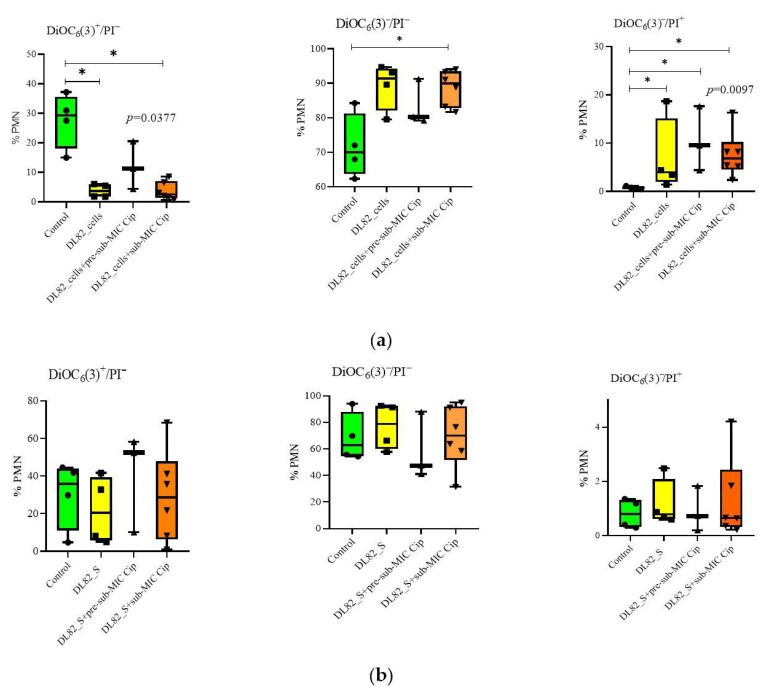
Neutrophil viability (DiOC_6_(3)^+^/PI^−^), early apoptosis (DiOC_6_(3)^−^/PI^−^), necrosis (DiOC_6_(3)^−^/PI^+^) during interactions of UPEC strain DL82 biofilm cells (**a**) and biofilm supernatants (S) (**b**) formed at pre-sub-MIC (0.002 mg/L) and sub-MIC (0.004 mg/L) of Cip. Control for cells (**a**) is 0.9% NaCl, control for supernatants (**b**) is LB. PMN—polymorphonuclear neutrophil, *—statistically significant difference from control at *p* < 0.05.

To determine whether *E. coli* grown in LB with different ciprofloxacin concentrations have different effects on human neutrophils (polymorphonuclear neutrophils, PMNs), assays for assessment of PMN viability/apoptosis/necrosis were performed. Dotplots of PMN subpopulations after interaction with UPEC strain DL82 are shown in Figure 3. PMN viability (DiOC_6_(3)^+^/PI^−^) was reduced by UPEC strain DL82 biofilm bacteria compared to the control regardless of the presence of ciprofloxacin in the medium (Figure 3a and Figure 4a). The number of PMNs in the early apoptosis stage (DiOC_6_(3)^−^/PI^−^) was increased by *E. coli* DL82 biofilm bacteria grown with the sub-MIC of ciprofloxacin (0.004 mg/L) (Figure 4a). Late apoptosis/necrosis of neutrophils (DiOC_6_(3)^−^/PI^+^) was enhanced compared to the control by *E. coli* DL82 biofilm bacteria grown with/without the pre-sub-MIC and sub-MIC of ciprofloxacin.

### 2.5. Neutrophil Size and Granularity After Interaction of UPEC Strain DL82 Cells and Supernatants of Biofilms Formed at Pre-Sub-MIC and Sub-MIC of Cip

**Figure 5:**
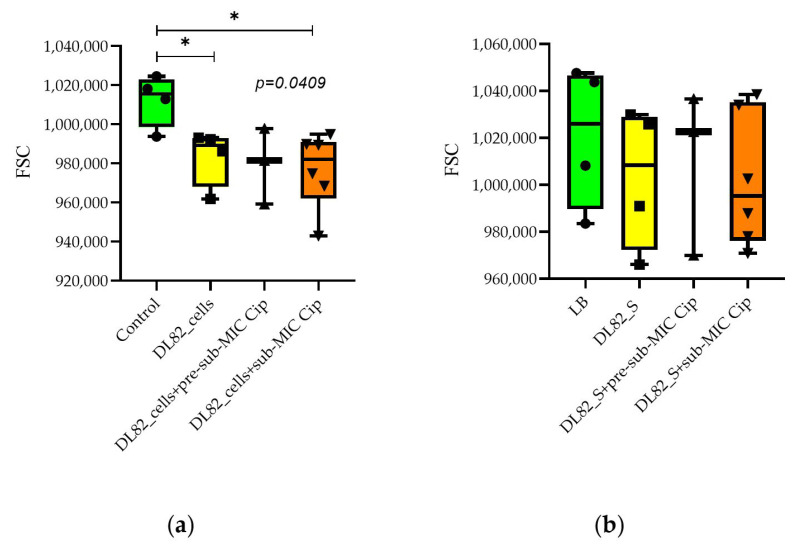
Neutrophil size upon interaction with biofilm cells (**a**) and supernatants (**b**) of UPEC strain DL82 formed at pre-sub-MIC (0.002 mg/L) and sub-MIC (0.004 mg/L) concentrations of Cip. G * S—supernatants. Control for cells (**a**) 0.9% NaCl, for supernatants (**b**) LB. FSC—forward scatter, *—statistically significant difference from control.

The size of neutrophils can be one of the indicators of their functional activity. As seen in Figure 5a, the *E. coli* DL82 strain biofilm bacteria grown without antibiotic in the medium and grown with sub-MIC of ciprofloxacin led to a decrease in the size of neutrophils, while the biofilm supernatant of *E. coli* DL82 (Figure 5b) did not affect the size of neutrophils compared to the control.

**Figure 6:**
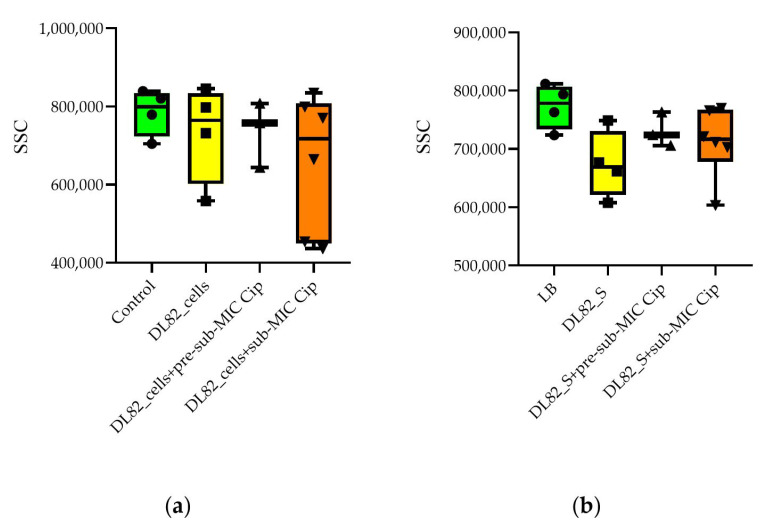
Neutrophil granularity upon interaction with biofilm cells (**a**) and supernatants (S) (**b**) of UPEC strain DL82 formed at pre-sub-MIC (0.002 mg/L) and sub-MIC (0.004 mg/L) of Cip. Control for cells (**a**) is 0.9% NaCl, control for supernatants (**b**) is LB. SSC—side scatter.

Neutrophil granularity was not altered by *E. coli* DL82 biofilm cells and supernatants grown with/without the pre-sub-MIC and sub-MIC of ciprofloxacin (Figure 6), however, the size of neutrophils tended to decrease when the concentration of ciprofloxacin was increased.

## 3. Discussion

Despite the limitations of fluoroquinolones due to the intrinsic characteristics of this antibiotic, they remain fundamental in treatment of UTIs thanks to their bioavailability and synergistic effects with other drugs. Moreover, fluoroquinolones play a critical role in eradicating biofilm-associated infections, as their antibiofilm activity is superior to that of beta-lactams and glycopeptides [24,25,26]. In this regard, we studied the interaction of biofilm bacteria and supernatants formed by ampicillin-resistant UPEC strain DL82, a strain with a high pathogenic potential, sensitivity to fluoroquinolones, and good ability for biofilm formation, in the presence of the sub-MIC and MIC of ciprofloxacin with PMNs, innate immunity factors.

Biofilm formation is a critical mechanism contributing to drug resistance in *E. coli*. A recent study showed that antibiotics, including cephalothin, ceftriaxone, ceftazidime, amikacin, and ciprofloxacin, with the exception of ampicillin, caused a significant reduction in biofilm biomass after 48 h [27]. In recent years, a variety of laboratory methods have been developed to evaluate antibiofilm therapy. The most significant endpoint parameter of cell susceptibility in a biofilm is the MBEC [21]. MBEC, the lowest antimicrobial concentration that will kill all the microorganisms in a biofilm in 24 h, including the persister cells, can be hundreds to thousands times higher than the MIC/MBC for the same antimicrobial–microorganism pair [20]. In our study, we found a significant increase in MBEC relative to MBC for ampicillin, chloramphenicol, and levofloxacin. However, we did not find a big difference between the concentrations that kill bacteria in plankton and biofilm forms for gentamicin and ciprofloxacin. Similar data were presented by Gastaldi Guerrieri et al. [28]. Since fluoroquinolones easily diffuse through the biofilm matrix and are able to kill cells in an attached state, this may be due to the formation of a large proportion of persister cells. Previously, Rafaque et al. [29] demonstrated that, with the exception of trimethoprim, other first-line antibiotics tested (levofloxacin, gentamicin, and ceftazidime) were ineffective in eliminating uropathogenic *E. coli* (UPEC) biofilms even at high concentrations. It seems biofilm formation for sensitive strains is a rather important form of survival under antibiotic pressure.

According to the literature, the sensitivity of bacteria to antibiotics (ampicillin) can increase at the adhesion stage for cells involved in the interaction between cell clusters during the formation of microcolonies. Perhaps, similar mechanisms can mediate the nature and massiveness of biofilm formation by bacteria in the presence of antibiotics. Moreover, exposure of *E. coli* to sub-inhibitory concentrations of antibiotics stimulated biofilm formation [30]. In our study, exposure to ampicillin, gentamicin, and chloramphenicol had inhibition effects on plankton growth and biofilm formation. However, the fluoroquinolone antibiotics (ciprofloxacin and levofloxacin) inhibited plankton growth but led to an increase in biomass.

The cellular response to the presence of an antibiotic is a complex process: a bacterial cell can change its growth rate, morphology, cellular composition, and other physiological characteristics depending on the mechanism of action and concentration of the antibiotic used. We found that the growth of *E. coli* DL82 biofilm biomass was stimulated by both the pre-sub-MIC and sub-MIC of ciprofloxacin, while an excess of ciprofloxacin concentration above the MIC led to a decrease in biofilm biomass and elongation of bacterial cells in the *E. coli* DL82 strain (Figure 3). It was previously shown that, when exposed to beta-lactams (amoxicillin–clavulanate, ceftriaxone), elongation of *E. coli* cells was observed, since the cell wall changed, due to inhibition of cell wall synthesis [31]. Ciprofloxacin treatment results in the formation of elongated cells or filamentous cells, mainly due to SOS-induced changes in *sulA* (formerly *sfiA* for suppressor of filamentation) functionality [32,33]. There is an increase in bacterial membrane dynamics and a rearrangement of inner membrane lipids [34]. The induction of the filamentous morphotype is key because it serves as a marker for cells that are stressed but remain metabolically active and viable. The increased survival of *E. coli* correlates with an increase in nucleoid length, as well as with the removal of misfolded proteins, an unexpected stress relief mechanism used by filamentous bacteria [35].

Most importantly, altered immune reactions are usually noted for biofilm and tumor microenvironments [36,37,38]. Altogether, there are crucial challenges for traditional antibiofilm and anticancer drugs to overcome these situations where pathogens and cancer cells survive and cause the onset of disorders [36,37]. UPEC can express a wide range of virulence factors like type I fimbriae, hemolysin, and transporters (sorbitol and cellulose) that cooperate in biofilm formation. Other virulence factor genes (iron acquisition systems, protectins, and mycelia) are expressed at high levels in biofilm-forming isolates and contribute to protection of the bacteria against host immune response cascades and cytokines [39,40]. The *E. coli* alpha-hemolysin (Hly) and cytotoxic necrotizing factor 1 (CNF1) are candidates for affecting neutrophil apoptosis or necrosis. The Hly enzyme is a member of the repeats-in-toxin family of pore-forming toxins [41]. CNF1 promotes an increase in the number of neutrophils and bacteria in infected bladder and kidney tissues, which mediates the subsequent development of inflammation and tissue damage [42]. Our study showed that, when interacting with bacteria in biofilms of *E. coli* DL82, which possesses the *hlyA* gene as well as the *cnf1* gene, the decrease in neutrophil viability was due to their necrosis (Figure 4). Apparently, the expression of UPEC virulence genes does not weaken in biofilms, which complicates the elimination of pathogens by innate immunity factors.

Findings observed in vitro showed that the human myeloid cell line HL60 (PMN-like cell line) increased expression of CD14 receptors [43] and thereby initiated apoptosis. According to recent data, CD14 is the major co-receptor for lipopolysaccharide detection on mouse dendritic cells (DCs) as a binding partner of FimH, the protein located at the tip of the type 1 fimbriae of *E. coli* [44]. In our studies, the UPEC strain DL82 possessed adhesin genes (*fimH*). We supposed that the interaction between biofilm bacterial cells with FimH adhesin on the tip of the fimbriae and CD14 of PMNs represents a potential target to interfere with persistent and recurrent urinary tract infections.

The stimulation of biofilm formation by sub-lethal doses of ciprofloxacin noted in our work may serve as additional evidence that antibiotics can change the virulence potential of bacteria, which is consistent with the data obtained in the literature [42]. In addition to direct contact of bacterial cells with neutrophils, the indirect effect of bacteria through extracellular metabolites (biofilm supernatants) on innate immune cells is important. As shown in our previous work, supernatants of commensal *E. coli* K12 decreased the viability of neutrophils; they did not alter the number of necrotic cells but increased the level of apoptosis [45].

The biofilms formed by commensal bacteria are partly activating neutrophils as part of a normal physiologic process, without exposing the host to the harmful effect of a fully active neutrophil response (para-inflammation) [46]. In this case, a subset of neutrophils are FSC^high^, characterized by larger size, low presence (or absence) of granules, presence of multiple vacuoles, and a significant euchromatin fraction, suggesting important changes in gene expression [47]. Opposite to para-inflammation is the fully activated proinflammatory state of PMNs. In this case PMNs are FSC^low^ cells, characterized by a smaller size, a considerably higher number of cytoplasmic granules, a low number of vacuoles, and a nucleus mainly constituted by heterochromatin [47]. As seen from Figure 6a, a FSC^low^ PMN subset appeared after addition of the cells of the virulent strain (*E. coli* DL82) without and with ciprofloxacin (sub-MIC). We supposed that FSC^low^ PMNs are unable to eliminate pathogens successfully. Some authors have reported that “bigger” neutrophils might correspond to cells experiencing an aging process or even might be cells going into apoptosis [48]. So, according to a previous suggestion, acute inflammation due to the FSC^low^ PMN subset may be modulated to para-inflammation (FSC^high^) that leads to chronicity of UTIs. In PMN cells, an SSC-A^high^ subtype is typically associated with the presence of numerous and dense granules with antibacterial properties [47]. The absence of a difference in the presence of neutrophil granularity when interacting with bacterial cells indirectly allows us to conclude that UPEC cells do not affect the functional characteristics of neutrophils. However, the tendency for a decrease in PMN granularity when exposed to supernatants allows us to conclude that, when interacting with bacterial exoproducts, neutrophils secrete the contents of the granules, which leads to the appearance of sub-populations with low granularity. This may be one of the mechanisms for the invasion of UPEC into the host. 

As for the tendency for the size of neutrophils to decrease in the presence of low concentrations of antibiotics, according to the literature, the uptake of fluorochinolons by PMNs is rapid, reversible, and non-saturable and is affected by environmental temperature, cell viability, and membrane stimuli. At therapeutic extracellular concentrations, gemifloxacin showed intracellular activity against *Staphylococcus aureus* [49]. Possibly, this mechanism is necessary for intracellular pathogen killing.

Thus, it should be noted that, in our work, an increase in the biomass of the VAG-possessing strain biofilm under the pre-sub-MIC and sub-MIC of ciprofloxacin did not change the fundamental picture of the interaction of neutrophils with bacterial cells from biofilms, which is an important fact in the treatment of recurrent infections. High biofilm biomass does not introduce a new mechanism of resistance or evasion of UPEC that would hinder the neutrophil’s ability to fight the infection. This information is valuable in developing treatment strategies for persistent and recurring infections where biofilms are a major factor.

## 4. Materials and Methods

### 4.1. Bacterial Strain

The UPEC strain DL82, which was kindly provided by Marjanca Starčič Erjavec, University of Ljubljana (Slovenia), was used in the study. Its known characteristics are given in Table 3.

### 4.2. Antibiotics

The following antimicrobial drugs were used in the work: ampicillin sodium salt, 91–102% (BioChemica, Sauerlach, Germany)—beta-lactam group; gentamicin (Dalkhimform, Khabarovsk, Russia)—aminoglycoside; levomycetin sodium succinate (chloramphenicol) (Biokhimik, Saransk, Russia)—an amphenicol antiseptic widely used in urology; ciprofloxacin (Sigma, Darmstadt, Germany) and levofloxacin (Fluka, Paris, France)—both fluoroquinolones. Two-fold dilutions of the drugs were carried out in Mueller–Hinton broth (Babia, Jinan, China).

### 4.3. Estimation of MIC, Sub-MIC, and Pre-Sub-MIC of Antibiotics

Overnight culture grown in Mueller–Hinton broth at 37 °C without aeration was standardized with fresh nutrient medium to a concentration of 10^6^ cells/mL. Then, 100 μL of the resulting suspension was added to the wells of a 96-well plate (Medpolymer, St Petersburg, Russia) with 100 μL of the studied concentration of the antimicrobial drug. Mueller–Hinton broth was used as a control. The plates were cultured at 37 °C for 24 h, after which the optical density (OD_600_) was measured. The MIC was considered as the minimum concentration of the drug at which planktonic growth of the culture was inhibited by 70% compared to the control. Growth inhibition by more than 70% was noticeable to the naked eye, which is used as an assessment of sensitivity to antibiotics according to the EUCAST system. Concentrations below MIC were considered as sub-MIC (0.5 × MIC) and even lower concentrations as pre-sub-MIC (0.25 × MIC).

### 4.4. Estimation of MBC

To assess the MBC, 5 μL of a mixture of bacterial suspension with dilutions of antimicrobial drugs was plated on Mueller–Hinton agar. The bactericidal concentration was considered to be the one at which there was no colony growth on the agar.

### 4.5. Estimation of MBEC

The MBEC assay was performed using 96-well microplates as described before [21]. Briefly, the UPEC strain was grown 24 h in Mueller–Hinton broth without aeration to form pre-formed biofilm. Then, the contents of the wells were gently discarded and washed three times with normal saline solution. At the same time, 100 μL serially two-fold diluted ampicillin (1024–5 × 10^4^ mg/L), gentamicin (1–128 mg/L), chloramphenicol (0.25–1024 mg/L), ciprofloxacin (0.004–1024 mg/L), and levofloxacin (0.004–2048 mg/L) in normal saline solution were added into the wells at the volume of 200 μL and incubated at 37 °C for 24 h without aeration. Finally, after discarding the contents of wells, they were washed three times with 0.9% NaCl, and 200 μL of fresh 0.9% NaCl was added to wells and cells were released from biofilms by ultrasound (Elma Ultrasonic 30S, Elma, Singen, Germany) by sonicating 5 times for 1 min. Then, 10 μL of bacterial suspension was cultured on Mueller–Hinton agar (Babia, China) at 37 °C for 48 h and the grown colonies were counted. The MBEC values for antibiotics were defined as the lowest amount of antibiotics required to kill 100% of the biofilm-embedded bacteria.

### 4.6. Estimation of Biofilm Biomass

To assess biofilm formation, overnight culture grown in Mueller–Hinton broth at 37 °C without aeration was standardized with fresh nutrient medium to a concentration of 10^6^ cells/mL. Then, 100 μL of the resulting suspension was added to the wells of a 96-well plate (Medpolymer, St Petersburg, Russia) with 100 μL of the studied concentration of the antimicrobial drug. LB broth was used as a control. The plates were cultured at 37 °C for 24 h, then the biofilms were washed three times with 0.9% NaCl, dried for 24 h at 20 °C, and stained with 1% crystal violet for 30 min. Then the biofilms were washed three times with 0.9% NaCl. Finally, 200 μL of 96% alcohol was added to the stained biofilm and the optical density (OD_580_) was determined on a Synergy H1 TM multiplate reader (BioTec, Waltham, MA, USA).

### 4.7. Microscopy of Biofilm

The structure of daily biofilms formed in LB broth without and with different concentrations of ciprofloxacin was visualized using the EVOS M5000 microscope.

### 4.8. Assessment of Neutrophil Viability/Apoptosis/Necrosis

Neutrophils from the peripheral blood of healthy men (*n* = 4) were isolated on a double Ficoll–Urografin gradient (1.077 g/mL and 1.112 g/mL). Volunteers provided written informed consent when donating blood in accordance with the rules of the Ethics Committee of the IEGM UB RAS (Protocol № 24/2, dated 4 July 2023). The viability of neutrophils was 97% in the trypan blue test.

To form biofilm UPEC bacterial cultures (10^6^ cells/mL) the DL82 strain was grown in 96-well polystyrene plates for 24 h in Luria–Bertani (LB) medium (Sigma-Aldrich, Saint Louis, MO, USA) at 37 °C statically without antibiotics (control) and with ciprofloxacin in different concentrations as mentioned in the legends of the figures.

The supernatants (biofilm supernatants) were collected from the wells and sterilized by filtration (pore diameter 0.22 μm; Corning, Kaiserslautern, Germany). Then, bacterial cells were released from biofilms by ultrasound (Elma Ultrasonic 30S, Elma, Singen, Germany) by sonicating 5 times for 1 min and resuspended in RPMI 1640 medium. Neutrophils (250 μL; 10^5^ cells/mL) were cultivated with UPEC biofilm cells (100 μL suspension, RPMI 1640 medium—control) or their biofilm supernatants (100 μL; 100 μL LB—negative control, 100 μL LB with antibiotic—positive control) for 1 h in RPMI medium in a CO_2_ incubator.

Neutrophil viability was determined using DiOC_6_(3) (Invitrogen, Waltham, MA, USA) and PI (Invitrogen, Waltham, MA, USA) dyes. Live neutrophils had the DiOC_6_(3)^+^/PI^−^ phenotype, cells in the early apoptosis and necrosis stages—DiOC_6_(3)^−^/PI^−^ and DiOC_6_(3)^−^/PI^+^, respectively [50].

### 4.9. Statistics

Each experiment/assay was performed 3–6 times with 4–6 technical repeats. Statistical processing of the obtained results was performed using Excel, Statistica 6.0. Data were presented as the mean and standard deviation (M ± m) or medians and quartiles. Morphological parameters of neutrophils were assessed by the medians and quartiles of forward (FSC) and side scatter (SSC). The significance of differences was determined by the Student and Mann–Whitney tests at *p* < 0.05. Kruskal–Wallis tests were performed with non-parametric data. Correlation analysis was performed using the Pearson test at a significance level of *p* < 0.05.

## Figures and Tables

**Table 1:** Minimum inhibitory concentration (MIC), minimum bactericidal concentration (MBC) of uropathogenic (UPEC) strain DL82 planktonic cells, and minimum biofilm eradication concentration (MBEC) of UPEC strain DL82 biofilm cells.

Antibiotic	MIC	MBC	MBEC
	mg/L	mg/L	mg/L
Ampicillin	8192	8192	5 × 10^4^
Gentamicin	4	16	32
Chloramphenicol	1	16	512
Ciprofloxacin	0.008	0.032	0.062
Levofloxacin	0.016	0.032	1

**Table 2:** Correlation between plankton growth (OD_600_) and biofilm formation (OD_580_) of UPEC strain DL82 in the presence of antibiotics.

Antibiotic (Concentration Range)	r ^1^	*p*
Ampicilin (0–8192 mg/L)	0.95	0.001
Gentamicin (0–16 mg/L)	0.84	0.072
Chloramphenicol (0–4 mg/L)	0.99	0.0002
Ciprofloxacin (0–0.016 mg/L)	0.76	0.085
Levofloxacin (0–0.032 mg/L)	0.26	0.566

^1^ Pearson correlation coefficient at *p* ≤ 0.05.

**Table 3:** Phenotypic and genotypic characteristics of UPEC strain DL82.

Characteristic	UPEC DL82
**Phenotypic**	
Biofilm, OD_580_	0.985
Bacteriocin production	– ^1^
**Genotypic**	
* tra*	–
* papC*	+
* sfaDE*	+
* afa/draBC*	–
* cnf1*	+
* hly A*	+
* iroN*	+
* ibeA*	–

^1^ –/+—absence/presence of the characteristic, respectively.

## Data Availability

The original contributions presented in this study are included in the article. Further inquiries can be directed to the corresponding authors.

## Abbreviations

The following abbreviations are used in this manuscript:

Cip	Ciprofloxacin
FSC	Forward Scatter
MIC	Minimum Inhibitory Concentration
MBC	Minimum Bactericidal Concentration
MBEC	Minimum Biofilm Eradication Concentration
PMN	Polymorphonuclear Neutrophil
SSC	Side Scatter
UPEC	Uropathogenic
UTI	Urinary Tract Infection
